# Integrin *β*8 Facilitates Macrophage Infiltration and Polarization by Regulating CCL5 to Promote LUAD Progression

**DOI:** 10.1002/advs.202406865

**Published:** 2024-11-13

**Authors:** Lei Song, Xi Yu, Yang Wu, Wenwen Zhang, Yu Zhang, Yanchi Shao, Zhenxin Hou, Chen Yang, Yue Gao, Yanbin Zhao

**Affiliations:** ^1^ Department of Internal Medical Oncology Harbin Medical University Cancer Hospital Harbin Heilongjiang 150081 China; ^2^ Department of Gynecological Oncology Harbin Medical University Cancer Hospital Harbin Heilongjiang 150081 China; ^3^ Department of Breast Surgery Harbin Medical University Cancer Hospital Harbin Heilongjiang 150081 China; ^4^ College of Bioinformatics Science and Technology Harbin Medical University Harbin Heilongjiang 150081 China

**Keywords:** CCL5, ITG*β*8, LUAD, macrophage, TME

## Abstract

The tumor microenvironment (TME) influences cancer progression and metastasis. Integrin *β*8 (ITG*β*8), a member of the integrin family, is upregulated in various cancers. In this study, it is determined as a key factor that mediates the interaction between lung adenocarcinoma (LUAD) cells and macrophages. Increased expression levels of ITG*β*8 are associated with increased numbers of CD163+ macrophages and poor prognosis in LUAD patients. The overexpression of ITG*β*8 in LUAD cells promotes the polarization of THP‐1 macrophages toward the M2 phenotype. In contrast, TCM (conditioned medium from the co‐culture system) from THP‐1 macrophages and ITG*β*8‐overexpressing A549 cells promoted the proliferation and invasion of A549 cells. Mechanistically, chemokine (C‐C motif) ligand 5 (CCL5) plays an important role in mediating ITG*β*8‐induced macrophage polarization, and the phosphoinositide 3‐kinase (PI3K)/AKT serine/threonine kinase (AKT)/interferon regulatory factor 9 (IRF9) pathway is involved in this process. Moreover, interleukin 8 (IL8) and interleukin 10 (IL10) produced by M2‐like macrophages regulate the expression of ITG*β*8 in LUAD cells through the spi‐1 proto‐oncogene (SPI1). This study elucidates the feedback mechanism of ITG*β*8 between LUAD cells and macrophages.

## Graphical abstract

Integrin β8 (ITG*β*8) acts as a crosstalk mediator between tumor cells and tumor‐associated macrophages (TAMs) in tumor microenvironment (TME). In short, ITG*β*8 is expressed in tumor cells and promotes the secretion of chemokine (C‐C motif) ligand 5 (CCL5), which binds to its receptor CCL5‐C‐C motif chemokine receptor 5 (CCR5) on macrophages, thus promoting macrophage polarization toward the M2 phenotype. On the other hand, M2‐like macrophages secrete cytokines to promote the expression of ITG*β*8 in tumor cells.

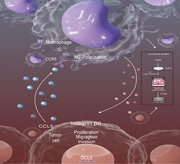

## 1 Introduction

Among all malignancies, lung cancer has the highest incidence and mortality rates worldwide.^[^ ^1^ ^]^ It can be broadly divided into small‐cell lung cancer (SCLC) and non‐small cell lung cancer (NSCLC), and NSCLC comprises >85% of all cases.^[^ ^2^ ^]^ The most common histological subtype of NSCLC is lung adenocarcinoma (LUAD) (40%).^[^ ^3^ ^]^ However, the five‐year overall survival (OS) rate of LUAD patients is less than 20%, despite significant advancements in multimodal treatment options, such as targeted therapy, immunotherapy, radiation, and noninvasive surgical resection, in recent decades.^[^ ^4^ ^]^ Therefore, to identify helpful targets for treatment, it is necessary to study the molecular mechanisms underlying the development of LUAD.

The tumor microenvironment (TME) influences cancer progression and metastasis and affects patient prognosis.^[^ ^5^ ^]^ Tumor‐associated macrophages (TAMs) are important components of the immune cell population in the TME,^[^ ^6^ ^]^ and an increase in M1‐like macrophages is generally believed to inhibit tumor development; However, M2‐like macrophages facilitate the growth and spread of tumors by secreting a variety of growth factors, cytokines, and proteases that promote angiogenesis, tumor cell proliferation, invasion, and metastasis and are correlated with a poor prognosis.^[^ ^7^, ^8^, ^9^ ^]^ Additionally, M2‐like macrophages can increase immune tolerance which can help tumors evade immune clearance.^[^ ^10^, ^11^, ^12^ ^]^ Thus, M2‐like macrophages are considered potential targets for adjuvant anticancer therapies, and recent therapeutic approaches targeting M2 polarization of TAMs have shown encouraging results.^[^ ^13^ ^]^ There is a complex crosstalk between macrophages and tumor cells. According to studies, macrophages regulate the malignant behavior of tumor cells. On the other hand, tumor cell‐derived chemokines, exosomes, and lactate regulate macrophage polarization and recruitment.^[^ ^14^, ^15^, ^16^ ^]^ Therefore, a more in‐depth analysis of the crosstalk between tumor cells and TAMs and determination of the underlying mechanisms are crucial for identifying novel treatment targets.

Integrins are a family of transmembrane glycoprotein adhesion molecules that can mediate cell‐extracellular matrix (ECM) interactions and activate various intracellular signaling pathways.^[^ ^17^ ^]^ Studies on the interaction between integrins and TAMs have been reported. Integrin αv*β*5 acts as a receptor on glioma stem cells and binds to transforming growth factor beta (TGFBI) secreted by TAM. Integrin αv*β*5 then activates the SRC proto‐oncogene, nonreceptor tyrosine kinase (Src) signaling pathway and the signal transducer and activator of transcription 3 (STAT3) pathway, which promotes tumor growth driven by glioblastoma (GBM) stem cells.^[^ ^18^ ^]^ In ovarian cancer, periostin (POSTN) interacts with integrin αv*β*3 and integrin αv*β*5 and regulates macrophage polarization through nuclear factor kappa B (NF‐kB).^[^ ^19^ ^]^ However, the role of ITG*β*8 in LUAD cells and TAMs has not yet been elucidated.

The phosphatidylinositol 3‐kinase/protein kinase‐B (PI3K/AKT) signaling pathway has been described as one of the most frequently disrupted signaling pathways in cancer and is critical for cell motility, growth, and survival.^[^ ^20^, ^21^ ^]^ As the main component of the downstream interferon pathway, interferon regulatory factor 9 (IRF9) not only plays a crucial role in the defense against viruses and inflammation^[^ ^22^, ^23^ ^]^ but also participates in angiogenesis^[^ ^24^ ^]^ and resistance to anticancer drugs.^[^ ^25^ ^]^ Furthermore, chemokines are important messengers that mediate the interaction between tumor cells and TAMs. According to previous reports, chordoma cells recruit and polarize TAMs by secreting chemokine (C‐C motif) ligand 5 (CCL5) to promote malignancy progression.^[^ ^26^ ^]^ On the contrary, CCL5 can be produced by TAMs and promote prostate cancer cell stemness and metastasis through the activation of *β*‐catenin/STAT3 signaling.^[^ ^27^ ^]^ Blocking the CCL5‐C‐C motif chemokine receptor 5 (CCR5) axis in the TME may represent a potential clinically viable strategy to inhibit the growth of human phyllodes breast tumors.^[^ ^28^ ^]^ Therefore, targeting the CCL5‐CCR5 axis and related pathways is hypothesized to be a novel therapeutic strategy for LUAD.

Here, we showed that ITG*β*8 is upregulated in LUAD and is significantly associated with a poor prognosis. ITG*β*8 promotes the progression of LUAD by regulating macrophage polarization and recruitment through activation of the PI3K/AKT/IRF9/CCL5 axis. Moreover, interleukin 8 (IL8) and interleukin 10 (IL10) produced by TAMs promote ITG*β*8 transcription in tumor cells through spi‐1 protooncogene (SPI1). This study enhances the understanding and provides new insights into the crosstalk mechanisms that drive macrophage polarization and the progression of LUAD.

## 2 Result

### 2.1 High ITG*β*8 Expression is Correlated with Poor Prognosis and an Increased Number of TAMs in LUAD

**Figure 1:**
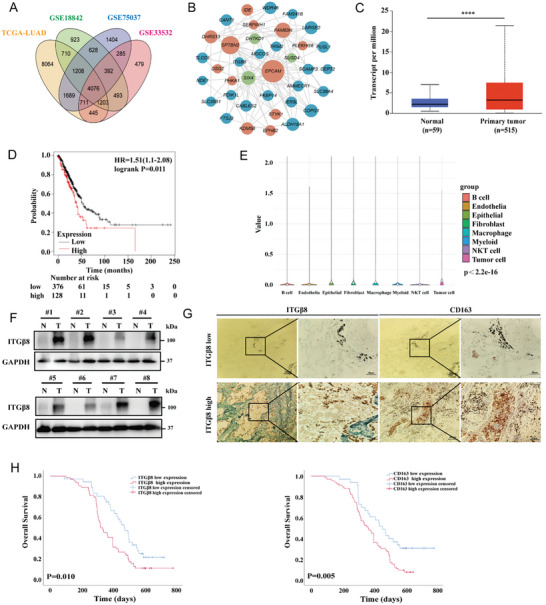
High ITG*β*8 expression is correlated with poor prognosis and an increased number of TAMs in LUAD. A) Venn diagram showing significant DEGs in the TCGA and GEO databases. B) Network of DEGs and immune‐ and EMT‐related genes. Blue indicates DEGs, and red and green indicate immune‐ and EMT‐related genes, respectively. C) ITG*β*8 expression in human LUAD tissues (n = 515) and normal tissues (n = 59) from the TCGA database. D) Kaplan–Meier curves showing the relationship between ITG*β*8 expression and OS in LUAD patients. E) The distribution of ITG*β*8 expression in the TME according to data from the GSE189357 dataset. F) ITG*β*8 expression levels in LUAD tumor (T) tissues and matched adjacent para‐carcinoma (N) tissues were examined by Western blotting. G) Representative images of IHC staining of ITG*β*8 and CD163 in human LUAD tissues. Magnification, 100× and 400×. The CD163 protein accumulated to higher levels in LUAD tissues with high expression of ITG*β*8. H) Survival curves displaying the relationships between ITG*β*8 or CD163 expression and OS in LUAD patients. (ns, not significant; *p < 0.05; **p < 0.01; ***p < 0.001; ****p < 0.0001).

Invasion and metastasis are among the most important biological characteristics of NSCLC and LUAD and are key factors that influence patient survival, whereas alterations in the TME are considered highly important in triggering these processes. In the present study, The Cancer Genome Atlas (TCGA) Research Network and Gene Expression Omnibus (GEO) databases were used to identify the differentially expressed genes (DEGs) between tumor tissues and normal tissues in the NSCLC and LUAD cohorts (Figure S1A, Supporting Information). The common DEGs among the four datasets are presented in a Venn diagram (**Figure** **1**A). In all, 4076 DEGs were identified. We subsequently constructed a coexpression network to screen the DEGs associated with immunity and epithelial‐mesenchymal transition (EMT). One of the most important networks is presented in Figure 1B, in which the blue bubbles represent DEGs and the red and green bubbles represent immune‐ and EMT‐related genes, respectively. Lines connecting two genes indicate possible regulatory effects. As a result, 22 DEGs related to immunity and EMT were screened. Notably, the role of ITG*β*8 in LUAD has been relatively less well studied than that of other members of the integrin family, and thus its involvement in tumor progression and the TME has not yet been fully elucidated.

The expression of ITG*β*8 in LUAD samples from the TCGA dataset was analyzed using the University of Alabama at the Birmingham Cancer data analysis portal (UALCAN), and the results indicated that ITG*β*8 is highly expressed in tumor tissues (N = 59, T = 515) (Figure 1C). Survival analysis of LUAD patients using the online Kaplan–Meier plotter software revealed that patients with high ITG*β*8 expression had significantly shorter OS than those with low ITG*β*8 expression (Figure 1D). To explore the role of ITG*β*8 in mediating the TME in LUAD, the distribution of ITG*β*8 expression was analyzed via single‐cell sequencing (scRNA‐seq). ITG*β*8 was most frequently expressed in tumor cells (Figure 1E), which suggests that this protein may indirectly affect infiltrating immune cells through tumor cells. Thus, we used TIMER to analyze the correlation between ITG*β*8 and the infiltration of various immune cell types in the TCGA‐LUAD cohort. Positive correlations were observed between ITG*β*8 and neutrophils (R = 0.35), macrophages (R = 0.25), and CD4+ T cells (R = 0.25) (Figure S1B, Supporting Information).

TAMs are TME components that are derived mainly from blood monocytes.^[^ ^29^ ^]^ Functionally, TAMs can be divided into classically activated (M1) and alternatively activated (M2) macrophages.^[^ ^30^ ^]^ Briefly, M1‐like macrophages inhibit tumor growth, whereas M2‐like macrophages promote tumor growth. In human tissue samples, Western blotting analysis revealed that ITG*β*8 was markedly overexpressed in 8 primary LUAD tissue samples compared with matched adjacent para‐carcinoma tissue samples (Figure 1F). IHC was used to analyze the protein levels of ITG*β*8 and CD163 in human LUAD tissues and to determine their clinical significance. The relationships of ITG*β*8 and CD163 expression with clinicopathologic features in 100 LUAD patients are provided in **Table** **1**. Patient information for all samples is provided in Table S1 (Supporting Information). As expected, CD163 protein expression levels were significantly greater in LUAD tissues with high expression of ITG*β*8 (Figure 1G). ITG*β*8 expression in LUAD was positively correlated with lymph node metastasis classification (p = 0.000) and T classification (p = 0.009), while CD163 expression in LUAD was positively correlated with T classification (p = 0.003) (Table 1). In addition, the survival curves demonstrated that patients with high ITG*β*8 and CD163 expression levels had shorter OS (*p* < 0.05, Figure 1H). These results suggest that ITG*β*8 is associated with poor prognosis in LUAD patients and may play an important role in regulating M2 polarization and macrophage infiltration.

**Table 1:** The relationship of ITG*β*8 expression and CD163 expression with clinicopathologic features in 100 LUAD patients.

Variables	Number of patients			Number of patients		
	ITG*β*8 low expression	ITG*β*8 high expression	p	CD163 low expression	CD163 high expression	p
Gender			0.149			0.137
Male	30	45		27	48	
Female	6	19		5	20	
Age (years)			0.423			0525
≤65	15	32		17	30	
>65	21	32		16	37	
Differentiation			0.295			0.518
Well/moderate	21	44		20	45	
poor	15	20		13	22	
T classification			0.009*			0.003*
T1	13	7		12	9	
T2	11	24		14	22	
T3 ∼ 4	12	33		7	36	
Lymph node Metastasis			0.000*			0.616
Negative	4	30		12	21	
Positive	32	34		21	46	

ITG*β*8 and CD163 scored ≥7, which was defined as a high expression; <7, represented a low expression. *p < 0.05 was considered statistically significant.

### 2.2 ITG*β*8 Plays a Crucial Role in the Polarization of Macrophage Toward the M2 Phenotype and Recruitment

**Figure 2:**
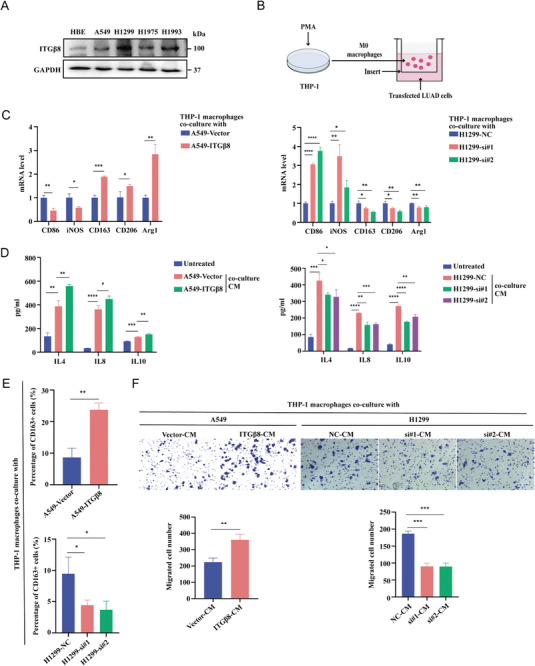
ITG*β*8 plays a crucial role in the polarization of macrophage toward the M2 phenotype and recruitment. A) Western blotting analysis of ITG*β*8 in HBE cells and four human LUAD cell lines. B) Schematic of an in vitro model in which THP‐1 macrophages were co‐cultured with transfected LUAD cells. C) qRT‐PCR was used to measure the expression of biomarkers of M1‐like and M2‐like macrophages in THP‐1 macrophages co‐cultured with LUAD cells. D) ELISA was used to measure the concentrations of IL4, IL8, and IL10 in the co‐culture system. E) Flow cytometry was used to explore the percentage of CD163+ THP‐1 macrophages co‐cultured with LUAD cells. F) Transwell assays were conducted to assess the effect of LUAD‐CM on the chemotactic ability of THP‐1 macrophages. (ns, not significant; *p < 0.05; **p < 0.01; ***p < 0.001; ****p < 0.0001).

To better understand the biological function of ITG*β*8, we assessed the expression of ITG*β*8 in a panel of four human LUAD cell lines and one normal human bronchial epithelial (HBE) cell line by Western blotting; the results revealed relatively high expression in A549, H1299, H1975, and H1993 cells compared with HBE cells (**Figure** **2**A). Therefore, A549 cells expressing an empty vector or ITG*β*8 were generated using a lentivirus expression system to construct a control group and an ITG*β*8‐overexpressing group, while H1299 and H1993 cells expressing a negative control (NC) or si‐ITG*β*8 were generated via transfection of small interfering RNA (siRNA) to construct a control group and an ITG*β*8‐knockdown group; these cell lines were used to explore the underlying molecular mechanisms, and changes in mRNA and protein expression were verified via quantitative reverse transcriptase PCR (qRT‐PCR) and Western blotting (Figure S1C, Supporting Information).

Based on the relationship between ITG*β*8 and macrophages, we further explored the mechanism by which ITG*β*8 regulates macrophage polarization using a co‐culture system (Figure 2B). THP‐1 cells were treated with 100 ng mL^−1^ phorbol 12‐myristate 13‐acetate (PMA) for 48 h to allow them to differentiate into M0 macrophages. qRT‐PCR revealed that CD68 expression was upregulated in these cells compared with untreated THP‐1 cells (Figure S1D, Supporting Information). Then, THP‐1‐derived macrophages were co‐cultured with tumor cells in which ITG*β*8 was overexpressed (A549 cells) or knocked down (H1299 and H1993 cells). Typical M1 phenotype biomarkers (CD86 and iNOS) and M2 phenotype biomarkers (CD163, CD206, and Arg1) were assessed via qRT‐PCR (Figure 2C; Figure S1E, Supporting Information). THP‐1 macrophages co‐cultured with ITG*β*8‐overexpressing cells presented increased CD163, CD206, and Arg1 levels and significantly decreased CD86 and iNOS levels. In contrast, the knockdown of ITG*β*8 significantly decreased CD163, CD206, and Arg1 levels and increased CD86 and iNOS levels in THP‐1 macrophages.

The cytokines related to the M2 phenotype were assessed via enzyme‐linked immunosorbent assay (ELISA) (Figure 2D; Figure S1F, Supporting Information). In our co‐culture system, interleukin 4 (IL4), IL8, and IL10 levels were significantly increased in the medium from THP‐1 macrophages co‐cultured with tumor cells overexpressing ITG*β*8, which indicates that more macrophages exhibited the M2 phenotype. Conversely, the concentrations of these cytokines in the supernatant of the ITG*β*8‐knockdown group were decreased. Flow cytometry revealed that co‐culture with ITG*β*8‐overexpressing cells increased the number of CD163+ cells that had differentiated from THP‐1 macrophages, whereas co‐culture with ITG*β*8‐knockdown cells decreased the number of CD163+ cells (Figure 2E; Figure S1G, Supporting Information).

Next, conditional medium (CM) from the ITG*β*8‐altered LUAD cell lines was added to the bottom of Transwell inserts for the macrophage chemotaxis assay. Interestingly, CM exerted chemotactic effects on THP‐1 macrophages, and these effects differed according to the ITG*β*8 expression status (overexpression or knockdown) of the cells (Figure 2F; Figure S1H, Supporting Information). Overall, these results support the hypothesis that the expression of ITG*β*8 in LUAD cells promotes macrophage recruitment and differentiation toward the M2 phenotype.

### 2.3 ITG*β*8 Promotes the Malignant Phenotype of LUAD Through M2‐Like Macrophages

**Figure 3:**
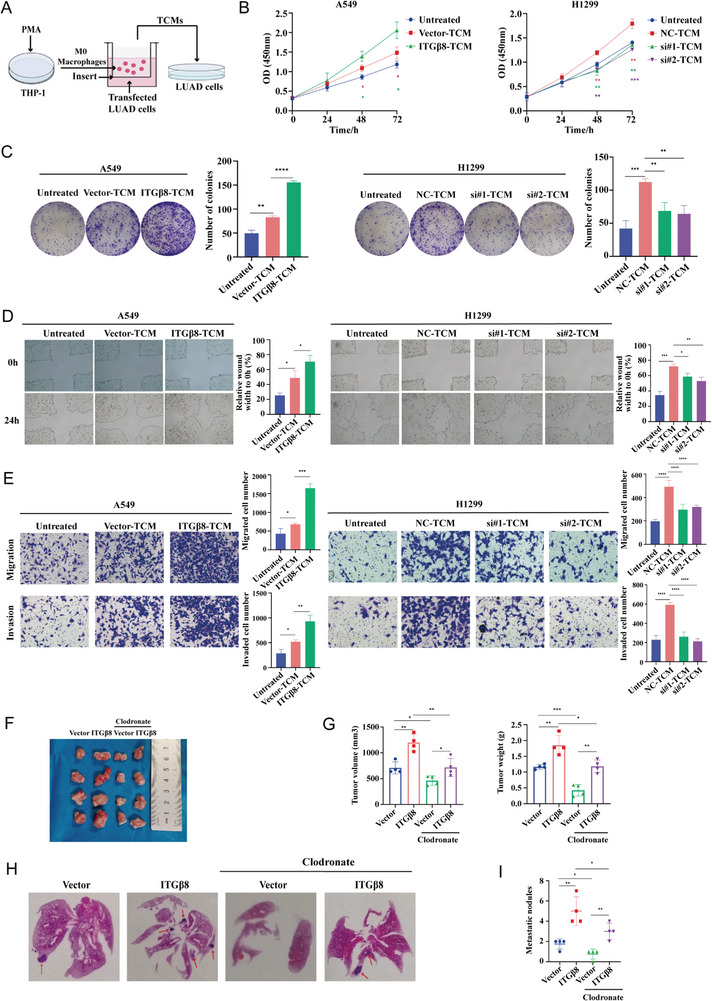
ITG*β*8 promotes the malignant phenotype of LUAD through M2‐like macrophages. A) Schematic of an in vitro model of LUAD cells treated with TCM collected from the co‐culture system. B,C) The effects of TCM on LUAD cell viability were analyzed via CCK‐8 (B) and colony formation (C) assays. D,E) The effects of TCM on the migration and invasion ability of LUAD cells were evaluated via wound healing (D), Transwell migration (upper panel), and invasion (lower panel) assays (E). F,G) Clodronate was injected into BALB/c nude mice to deplete mouse macrophages. Stably transfected A549‐Vector or A549‐ITG*β*8 cells were subcutaneously injected into the right axillary regions of nude mice (n = 4). The resulting tumors were harvested (F), and the tumor volume (left panel) and tumor weight (right panel) were measured (G). H,I) Tail vein injection of A549‐Vector or A549‐ITG*β*8 cells into nude mice treated with or without clodronate (n = 4). H&E‐stained images of lung tissues (H), and the number of metastatic nodules (I) are shown. (ns, not significant; *p < 0.05; **p < 0.01; ***p < 0.001; ****p < 0.0001).

TCM was obtained from a co‐culture system of ITG*β*8‐modified LUAD cell lines and THP‐1 macrophages and then added to tumor cells for subsequent functional experiments (**Figure** **3**A). CCK‐8 and colony formation assays revealed that A549‐ITG*β*8‐TCM promoted tumor cell proliferation, whereas H1299‐siITG*β*8‐TCM and H1993‐siITG*β*8‐TCM significantly inhibited tumor cell proliferation (Figure 3B,C; Figure S2A,B, Supporting Information). Furthermore, we aimed to elucidate the effects of M2‐like macrophages mediated by ITG*β*8 on LUAD metastasis via wound healing and Transwell migration assays (Figure 3D,E; Figure S2C,D, Supporting Information). TCM was added to the tumor cells or placed on the bottom of Transwell inserts. The results revealed improved migration ability of tumor cells treated with A549‐ITG*β*8‐TCM, whereas the opposite effect was observed with H1299‐siITG*β*8‐TCM and H1993‐siITG*β*8‐TCM. A consistent effect was observed in the Transwell invasion assay (Figure 3E; Figure S2D, Supporting Information). These findings demonstrate that ITG*β*8 affects the malignant behavior of LUAD cells by regulating the M2 polarization of macrophages in vitro.

To investigate the role of ITG*β*8 in controlling the interaction between macrophages and tumor cells, we used several animal models of cancer. First, clodronate‐containing liposomes were injected into BALB/c nude mice to deplete mouse macrophages. A549‐Vcetor cells and A549‐ITG*β*8 cells were then subsequently injected into BALB/c nude mice treated with or without clodronate‐containing liposomes. As shown in Figure 3F,G, in the group treated without clodronate, the tumors formed by the injection of A549‐ITG*β*8 cells were larger than those formed by the injection of A549‐Vector cells, in terms of size, volume, and weight. However, compared with mice injected with A549‐ITG*β*8 cells, nude mice injected with A549‐ITG*β*8 cells and treated with clodronate had smaller tumors, indicating that the depletion of macrophage counteracted the tumor‐promoting effect of ITG*β*8.

Furthermore, we generated an in vivo tail vein‐lung metastasis model in BALB/c nude mice using the same groups of cells. Seventy days later, the mice were sacrificed for analysis of tumor cell lung metastasis. H&E staining revealed that the overexpression of ITG*β*8 significantly promoted lung metastasis compared with the control group, whereas the depletion of macrophages suppressed lung metastasis (Figure 3H,I). Collectively, these findings strongly support our hypothesis that ITG*β*8 plays an oncogenic role in LUAD progression via macrophages in vivo.

### 2.4 ITG*β*8 Induces the Polarization and Chemotactic Activity of THP‐1 Macrophages Through CCL5

**Figure 4:**
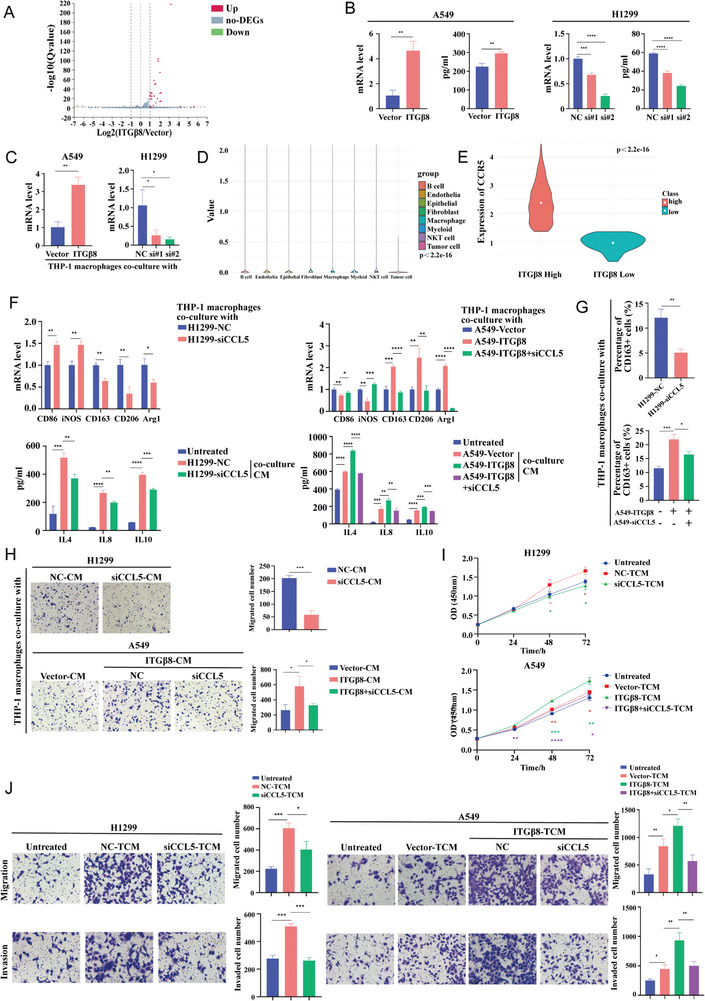
ITG*β*8 induces the polarization and chemotactic activity of THP‐1 macrophages via CCL5. A) Volcano plot showing DEGs in the A549‐Vector and A549‐ITG*β*8 groups. B) qRT‐PCR and ELISA were used to quantify the regulation of CCL5 mRNA and secretion levels induced by ITG*β*8. C) qRT‐PCR analysis of the expression of CCR5 in THP‐1 macrophages co‐cultured with LUAD cells. D) The expression of CCR5 in the TME according to data from the GSE189357 dataset. E) In ITG*β*8^high^ tissues, CCR5 is highly expressed (TCGA‐LUAD). F) M1‐like and M2‐like macrophage biomarker levels were measured via qRT‐PCR (upper panel); IL4, IL8, and IL10 secretion levels were quantified via ELISA (lower panel). G) Flow cytometry was used to explore the percentage of CD163+ THP‐1 macrophages co‐cultured with LUAD cells. H) Transwell assays were used to assess the effect of LUAD‐CM on the chemotactic ability of THP‐1 macrophages. I) The effect of TCM on LUAD cell viability was analyzed via a CCK‐8 assay. J) The effects of TCM on the migration and invasion ability of LUAD cells were assessed via Transwell migration (upper panel) and invasion assays (lower panel). (ns, not significant; *p < 0.05; **p < 0.01; ***p < 0.001; ****p < 0.0001).

We further elucidated the mechanism by which ITG*β*8 regulates M2 polarization and the infiltration of macrophages. RNA sequencing (RNA‐seq) was performed in A549 cells in which ITG*β*8 was overexpressed. From the results, we identified 30 DEGs, which were defined as those with log2|FC|≥ 1 and Q<0.05, that are shown in a volcano plot (**Figure** **4**A). Furthermore, Gene Ontology (GO) and Kyoto Encyclopedia of Genes and Genomes (KEGG) functional process analyses suggested that ITG*β*8 is involved in a variety of cellular processes, including infectious diseases, immune systems, and signal transduction (Figure S3A, Supporting Information).

Among the numerous DEGs (Table S2, Supporting Information), we elected to focus on CCL5. Chemokines and cytokines are the major factors that regulate macrophage polarization and recruitment.^[^ ^31^, ^32^ ^]^ Specifically, the CCL5‐CCR5 axis plays important roles in macrophage recruitment, polarization, and tumor progression. We further verified that the mRNA and concentration levels of CCL5 were increased after overexpression and decreased by ITG*β*8 knockdown in LUAD cell lines (Figure 4B; Figure S3B, Supporting Information). Subsequently, CCR5 expression was detected in THP‐1 macrophages after co‐culture and was upregulated by ITG*β*8 overexpression and downregulated by ITG*β*8 knockdown (Figure 4C; Figure S3C, Supporting Information). Moreover, CCR5, a CCL5 receptor, was relatively highly expressed in macrophages in the scRNA‐seq dataset (Figure 4D). In addition, analysis of the TCGA‐LUAD cohort revealed that CCR5 was upregulated in the group with high ITG*β*8 expression (Figure 4E). These findings suggest that ITG*β*8 expression induces tumor cells to secrete more CCL5 and thereby bind more macrophages, which promotes their polarization toward the M2 phenotype.

Next, we knocked down CCL5 in H1299 and H1993 cells or directly added recombinant human CCL5 (rhCCL5) to the culture medium of THP‐1 macrophages. According to the qRT‐PCR and ELISA results, THP‐1 macrophages co‐cultured with H1299‐siCCL5 and H1993‐siCCL5 cells exhibited downregulation of M2 biomarkers and decreased secretion levels of IL4, IL8, and IL10, whereas THP‐1 macrophages treated with rhCCL5 exhibited increased expression of M2 biomarkers and increased secretion of IL4, IL8, and IL10 (Figure 4F; Figure S3D,E, Supporting Information). Similarly, the number of CD163+ THP‐1 macrophages co‐cultured with CCL5‐knockdown cells was lower than that in the control group, while rhCCL5 increased the number of CD163+ THP‐1 macrophages, as determined by flow cytometry (Figure 4G; Figure S3F, Supporting Information). We subsequently knocked down CCL5 in A549‐ITG*β*8 cells for blocking experiments. Importantly, compared with ITG*β*8 overexpression, CCL5 knockdown in A549‐ITG*β*8 cells partially abrogated the ability of ITG*β*8 to promote the polarization of THP‐1 macrophages to the M2 phenotype, as indicated by decreased expression of CD163, CD206, and Arg1, decreased secretion of IL4, IL8, and IL10, and a decreased number of CD163+ THP‐1 macrophages (Figure 4F,G). Moreover, in the chemotaxis assay, H1299‐siCCL5‐CM and H1993‐siCCL5‐CM decreased the number of migrating THP‐1 macrophages, and the addition of rhCCL5 increased the number of migrating THP‐1 macrophages, whereas the knockdown of CCL5 in A549‐ITG*β*8 cells reversed the chemotaxis of THP‐1 macrophages induced by ITG*β*8 (Figure 4H; Figure S3G, Supporting Information).

Functional assays were conducted to determine the role of CCL5 in the ITG*β*8‐induced interaction between macrophages and LUAD progression. We observed that the proliferation ability of H1299 and H1993 cells treated with H1299‐siCCL5‐TCM and H1993‐siCCL5‐TCM was decreased, whereas rhCCL5‐TCM resulted in a significant increase in the proliferation of A549 cells compared with that of control cells (Figure 4I; Figure S4A, Supporting Information). Additionally, CCL5 knockdown reversed the effect of A549‐ITG*β*8‐TCM on the proliferation of A549 cells (Figure 4I). Transwell assays further revealed that H1299‐siCCL5‐TCM and H1993‐siCCL5‐TCM resulted in significantly reduced migration and invasion ability. In contrast, rhCCL5‐TCM enhanced the migration and invasion ability of A549 cells (Figure 4J; Figure S4B, Supporting Information). Moreover, TCM from CCL5 knocking down based on ITG*β*8 overexpression in A549 cells decreased the number of migratory and invasive tumor cells (Figure 4J). Overall, our results reveal that the effect of ITG*β*8 on the crosstalk between tumor cells and macrophages occurs via the regulation of CCL5.

### 2.5 ITG*β*8 Regulates CCL5 via PI3K/AKT/IRF9 Signaling

**Figure 5:**
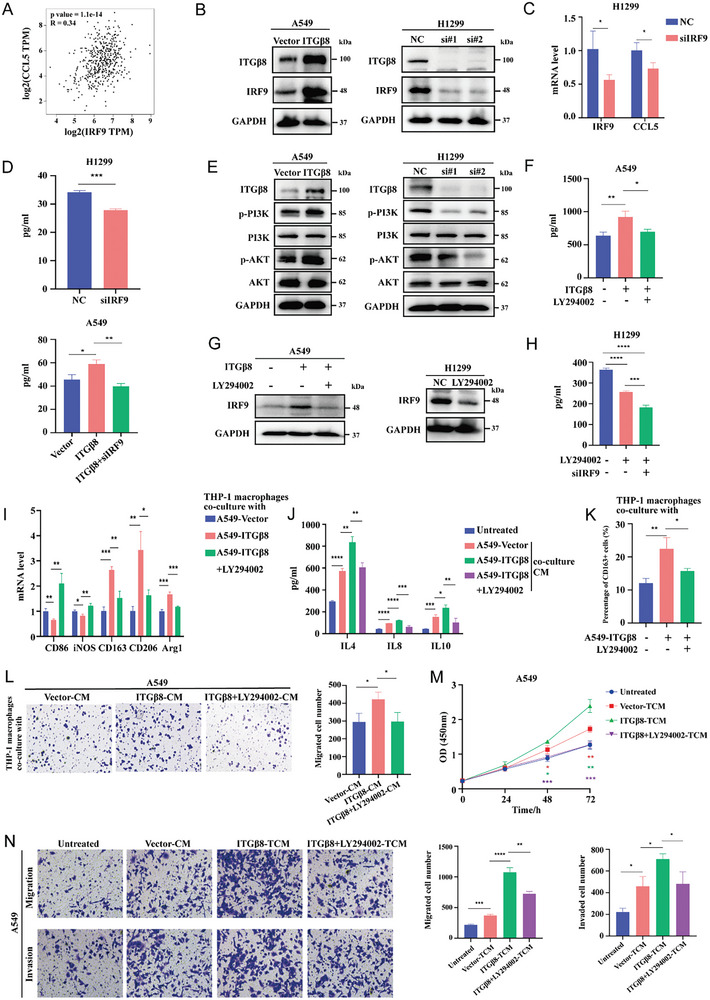
ITG*β*8 regulates CCL5 via PI3K/AKT/IRF9 signaling. A) Correlation analysis of IRF9 and CCL5 was performed via GEPIA2. B) The expression level of IRF9 in LUAD cells was measured by Western blotting. C) The expression of IRF9 and CCL5 mRNA levels was measured via qRT‐PCR in H1299‐siIRF9 cells. D) ELISA was used to assess the level of CCL5 secretion by H1299 and A549 cells. E) Western blotting was used to measure the levels of key signal transduction proteins. F) The effect of LY294002 (50 µM) on the secretion of CCL5 was evaluated via ELISA. G) Western blotting was used to assess the effect of LY294002 on the expression level of IRF9 in LUAD cells. H) The effects of LY294002 and siIRF9 on the level of CCL5 secretion were measured via ELISA. I) M1‐like and M2‐like macrophage biomarker expression was measured via qRT‐PCR. J) IL4, IL8, and IL10 secretion were quantified via ELISA. K) Flow cytometry was used to explore the percentage of CD163+ THP‐1 macrophages co‐cultured with LUAD cells treated with or without LY294002. L) Transwell assays were conducted to assess the effect of LY294002 on the chemotactic ability of THP‐1 macrophages induced by ITG*β*8. M) The proliferation ability of A549 cells was analyzed via a CCK‐8 assay. N) The migration and invasion abilities of A549 cells were assessed via Transwell migration (upper panel) and invasion (lower panel) assays. (ns, not significant; *p < 0.05; **p < 0.01; ***p < 0.001; ****p < 0.0001).

Our results showed that ITG*β*8 regulates the mRNA and protein levels of CCL5. Therefore, we searched for transcription regulatory factors, including interferon regulatory factor 7 (IRF7), basic leucine zipper transcription factor, ATF‐like 2 (BATF2), IRF9, and SMAD family member 9 (SMAD9), in our RNA‐seq data. The relationship between IRF9 and CCL5 needs to be further explored. Thus, we investigated CCL5 as a potential target of IRF9, and a positive correlation was found between CCL5 and IRF9 in LUAD based on Gene Expression Profiling Interactive Analysis 2 (GEPIA2) (**Figure** **5**A). We verified by Western blotting whether the expression of IRF9 is regulated by ITG*β*8. As expected, the protein levels of IRF9 increased after ITG*β*8 overexpression but decreased when ITG*β*8 was knocked down (Figure 5B). Moreover, the knockdown of IRF9 reduced the mRNA level and concentration of CCL5 in H1299 cells (Figure 5C,D), and the secretion of CCL5 induced by ITG*β*8 in A549 cells was partially abolished by the knockdown of IRF9 (Figure 5D), which indicates that IRF9 mediates the induction of CCL5 by ITG*β*8.

The KEGG pathway analysis performed via the TISIDB database revealed that ITG*β*8 was associated with common signaling pathways in LUAD, such as those involving P13K/AKT, focal adhesion and ECM‐receptor interactions, cell adhesion molecules (CAMs) and the regulation of the actin cytoskeleton (Figure S4C, Supporting Information). The PI3K/AKT pathway is one of the most frequently activated signaling pathways in human cancers, as it promotes tumor cell survival, proliferation, metabolism, invasion, and angiogenesis.^[^ ^20^, ^33^ ^]^ Thus, in the present study, we investigated the changes in the expression of these signaling proteins. The upregulation of ITG*β*8 activated the phosphorylation of key PI3K/AKT proteins in A549 cells, whereas the downregulation of ITG*β*8 inhibited these proteins in H1299 cells (Figure 5E). We also exposed ITG*β*8‐overexpressing A549 cells to LY294002 (a PI3K/AKT pathway inhibitor, 50 µM). Notably, treatment with LY294002 reversed the increase in CCL5 secretion observed in the ITG*β*8‐overexpressing A549 cells (Figure 5F). Next, we examined whether IRF9 is regulated by the ITG*β*8‐PI3K/AKT axis. As expected, IRF9 expression was reduced in H1299 cells treated with LY294002, and the addition of LY294002 reversed the upregulation of IRF9 induced by ITG*β*8 in A549 cells (Figure 5G). Moreover, according to the ELISA results, LY294002 treatment and IRF9 knockdown more significantly inhibited CCL5 secretion in H1299 cells than treatment with LY294002 alone (Figure 5H). Therefore, ITG*β*8 regulates CCL5 by activating the PI3K/AKT/IRF9 signaling pathway.

As shown in Figure 5I–K, the M2‐like polarization of THP‐1 macrophages was significantly increased when they were co‐cultured with A549‐ITG*β*8 cells, and this effect was partially abolished by LY294002. The chemotaxis of THP‐1 macrophages was promoted when the cells were incubated with A549‐ITG*β*8‐CM, and treatment with LY294002 partially reversed this effect (Figure 5L). Similarly, the LY294002 treatment reversed the increase in the proliferation, migration, and invasion of A549 cells induced by A549‐ITG*β*8‐TCM in the CCK‐8 and Transwell assays (Figure 5M,N).

In addition, we elucidate the roles of PI3K/AKT/IRF9 and CCL5 in the in vivo macrophage polarization induced by ITG*β*8. The qRT‐PCR results indicate that compared to the control group, LY294002, shIRF9, and shCCL5 decreased the expression levels of CD163, and Arg1 respectively, and increased the expression level of CD86. ITG*β*8 enhances the expression levels of CD163 and Arg1 and reduces the expression level of CD86. However, LY294002, shIRF9, and shCCL5 each reverse the M2 polarization capability of macrophages induced by ITG*β*8 to varying extents, as shown in Figure S4D, Supporting Information. These results indicate that ITG*β*8 regulates macrophage differentiation toward the M2 phenotype both in vitro and in vivo at least partially through the PI3K/AKT/IRF9/CCL5 axis.

### 2.6 ITG*β*8 Facilitates LUAD Progression In Vivo

**Figure 6:**
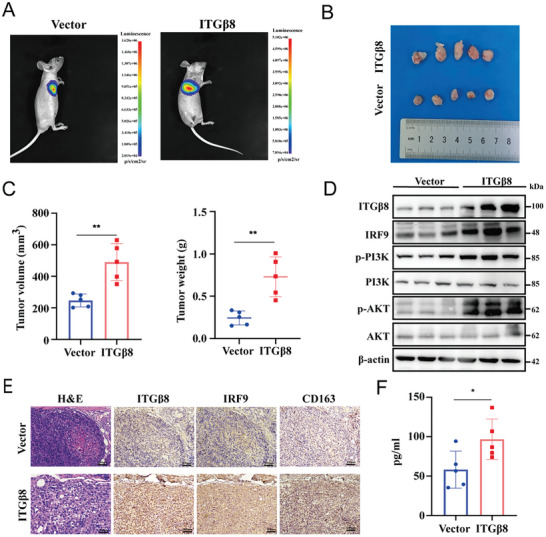
ITG*β*8 facilitates LUAD progression in vivo. Stably transfected Vector/ITG*β*8 cells (A549 cells expressing luciferase) were subcutaneously injected into the armpit regions of nude mice (n = 5). A) Tumor formation was monitored via bioluminescence imaging. B) Images of resulting tumors on Day 35. C) Tumor volume (left panel) and tumor weight (right panel) in the different groups. D) Western blotting analysis of the expression of the indicated markers in protein extracts obtained from harvested tumors. E) H&E and IHC staining were used to confirm the expression of the indicated markers in the two groups of tumor samples. Magnification, 100×. F) Apex cordis blood was extracted for ELISA to determine the concentration of CCL5. (ns, not significant; *p < 0.05; **p < 0.01; ***p < 0.001; ****p < 0.0001).

To investigate the potential functions of ITG*β*8 in the progression of LUAD in vivo, A549 cells were stably transduced with an empty vector or ITG*β*8 via a lentiviral system that contained luciferase. The cells were subcutaneously injected into the right axilla of BALB/c nude mice. Tumor growth was monitored for 1–5 weeks after implantation before removal for analysis. According to representative bioluminescence images, luciferase activity was significantly greater in the ITG*β*8‐overexpressing group than in the control group (**Figure** **6**A). The tumor size, volume, and weight exhibited the same trend (Figure 6B,C).

The overexpression of ITG*β*8 increased the expression of IRF9, p‐PI3K, and p‐AKT, as shown by Western blotting (Figure 6D). H&E and IHC revealed that the protein levels of IRF9 and CD163 were greater in the tumor tissues from the ITG*β*8‐overexpressing group than in those from the control group (Figure 6E). Finally, before the nude mice were sacrificed, apex cordis blood was extracted for ELISA, and the concentration of CCL5 in the plasma of mice injected with ITG*β*8‐overexpressing cells was greater than that in the plasma of control mice (Figure 6F). Thus, ITG*β*8 activates the PI3K/AKT/IRF9/CCL5 axis, which promotes LUAD progression in vivo.

### 2.7 Induction of ITG*β*8 Expression in Tumor Cells by M2‐Like Macrophages

**Figure 7:**
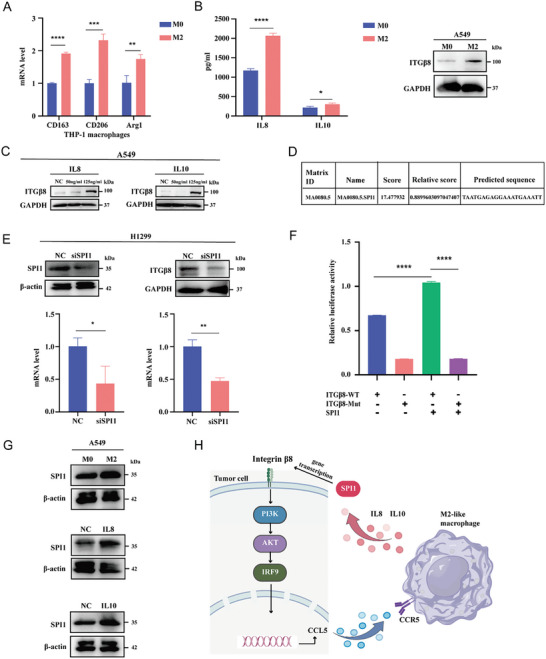
Induction of ITG*β*8 expression in tumor cells by M2‐like macrophages. A) M2‐like macrophage biomarkers were detected in THP‐1 macrophages treated with IL4 via qRT‐PCR. B,C) The concentration of IL8 and IL10 in M0/M2‐CM were measured via ELISA (left panel), Western blotting was used to measure the expression of ITG*β*8 in A549 cells treated with M0/M2‐like macrophage‐CM (B, right panel) or IL8/IL10 (100 ng mL^−1^) (C). D) Prediction of the binding site of SPI1 and ITG*β*8 promoters via JASPAR. E) Western blotting (upper panel) and qRT‐PCR (lower panel) were used to determine the effect of SPI1 knockdown on the expression of ITG*β*8. F) Relative luciferase activity in HEK293T cells after cotransfection of plasmids (pcDNA3.1) carrying the ITG*β*8 promoter (WT or Mut) with or without an SPI1‐overexpressing construct. G) Western blotting was used to measure the expression of SPI1 in A549 cells treated with M0/M2‐like macrophage‐CM (upper panel) or IL8 (middle panel) and IL10 (lower panel). H) Schematic model of the role of ITG*β*8 in LUAD and macrophage crosstalk. Some of the materials are sourced from FigDraw. (ns, not significant; *p < 0.05; **p < 0.01; ***p < 0.001; ****p < 0.0001).

To explore the effect of M2‐like macrophages on tumor cells, we investigated whether ITG*β*8 expression in tumor cells is induced by M2‐like macrophages. The THP‐1 cells were treated with PMA to induce their transformation into M0 macrophages, which were further polarized to the M2 phenotype with IL4. The polarization of macrophages was confirmed by qRT‐PCR (CD163, CD206, and Arg1) (**Figure** **7**A). CMs from M0/M2 macrophages were collected to detect the secretion levels of IL8 and IL10 via ELISA and for the culture of A549 cells in subsequent experiments (Figure 7B). Western blotting analysis revealed that CM from M2‐like macrophages induced ITG*β*8 expression in A549 cells (Figure 7B). TAMs secrete many cytokines, including IL4, IL8 and IL10. Therefore, we added 50 ng mL^−1^ and 125 ng mL^−1^ IL8 and IL10 to A549 cells. The results confirmed that IL8 and IL10 increased the protein levels of ITG*β*8 in A549 cells (Figure 7C), which indicates an interaction between macrophages and tumor cells.

To further elucidate the mechanism by which M2‐like macrophages induce ITG*β*8, we predicted the upstream regulatory transcription factors (TFs) of ITG*β*8 via the Database of Human Transcription Factor Targets (hTFtargets) and obtained 20 candidate TFs. GEPIA2 was used to analyze the correlation between ITG*β*8 and these TFs. Notably, ETS proto‐oncogene 1, transcription factor (ETS1), RELA proto‐oncogene, NF‐kB subunit (RELA), RE1 silencing transcription factor (REST), SMAD family member 3 (SMAD3), and SPI1 were significantly positively correlated with ITG*β*8 (Table S3, Supporting Information). We subsequently predicted the binding sites of these TFs to the ITG*β*8 promoter via JASPAR, and of all the TFs, SPI1 had the highest binding score. Therefore, SPI1 was identified as the most likely candidate for mediating the transcription of ITG*β*8. The binding site for SPI1 and the promoter of ITG*β*8 is shown in Figure 7D. As expected, the knockdown of SPI1 in H1299 cells markedly decreased the ITG*β*8 mRNA and protein levels (Figure 7E). Subsequent dual‐luciferase reporter analysis indicated that the luciferase activity of the reporter containing the wild‐type binding sites was induced by the ectopic expression of SPI1 (Figure 7F). The sequence of ITG*β*8 promoter‐WT and ITG*β*8 promoter‐MUT in the dual‐luciferase reporter analysis is provided in Table S4 (Supporting Information). We next validated the induction of SPI1 by M0/M2‐CM, IL8, and IL10 (Figure 7G). Overall, we confirmed that M2‐like macrophages induce the expression of SPI1 on tumor cells through IL8/IL10 to promote ITG*β*8 transcription.

Finally, this study shows that by regulating the PI3K/AKT/IRF9 pathway, ITG*β*8 promotes the secretion of CCL5 in tumor cells, thereby promoting the M2 polarization of macrophages and tumor progression. In contrast, the M2‐like macrophage‐derived cytokines IL8 and IL10 promote the expression of ITG*β*8 on tumor cells, and SPI1 mediates this effect (Figure 7H).

## 3 Discussion

The high morbidity and mortality rates of LUAD indicate the high degree of clinical need for the discovery of new effective therapeutic targets. According to previous reports, ITG*β*8 plays an important pro‐cancer role in various cancers. In NSCLC, circ‐0017956 promotes the proliferation and metastasis of NSCLC cells by regulating the miR‐515‐5p/ITG*β*8 axis.^[^ ^34^ ^]^ In bladder carcinoma, ITG*β*8 regulates the phosphorylation of Y box binding protein 1 (YBX1) and activates BCL2 apoptosis regulator (BCL2), which leads to drug resistance.^[^ ^35^ ^]^ However, research on the role of ITG*β*8 in the crosstalk between tumor cells and the TME in LUAD is still limited.

In this study, the analysis of the scRNA‐seq dataset revealed a relationship between ITG*β*8 and infiltrating immune cells in the TME. A positive correlation between ITG*β*8 expression and macrophage infiltration as well as CD163, CD206, and Arg1 expression indicates that ITG*β*8 is a key factor in the regulation of TAMs. Increasing evidence suggests that TAMs, important components of the TME, interact with tumor cells and play a key role in tumor progression. In colorectal cancer (CRC), cytoplasmic polyadenylation element binding protein 3 (CPEB3) inhibits interleukin 6 receptor (IL6R)/STAT3 signaling by binding to IL6R mRNA in CRC cells; this regulates the secretion of chemokine (C‐C motif) ligand 2 (CCL2), the polarization of TAMs, and the proliferation and invasion of CRC cells by inhibiting TAM‐derived interleukin 6 (IL6).^[^ ^36^ ^]^ In addition, triggering receptor expressed on myeloid cell‐2 (TREM2)‐positive TAMs accumulate in NSCLC and impair CD8+ T‐cell function, which contributes to poor clinical outcomes.^[^ ^37^ ^]^ Furthermore, the IL6‐STAT3‐CCAAT enhancer binding protein beta (C/EBP*β*)‐IL6 positive feedback loop in TAMs promotes EMT and metastasis in LUAD.^[^ ^38^ ^]^ Thus, the pro‐cancer behavior of TAMs makes them attractive therapeutic targets. A deeper exploration of the interaction between LUAD cells and TAMs should yield interesting results.

As expected, ITG*β*8 is highly expressed in LUAD tissues and cell lines, and CD163 is upregulated in tissues with high expression of ITG*β*8. Both of these factors are associated with poor prognosis in LUAD patients. In vitro, the expression of ITG*β*8 in LUAD cells promoted macrophage polarization toward the M2 phenotype. Moreover, TCM from the co‐culture system significantly altered the proliferation, migration, and invasion of LUAD cells. In vivo, the depletion of macrophages reduced the size and number of metastatic tumors induced by overexpression of ITG*β*8. These findings indicate that ITG*β*8 promotes the progression of LUAD through M2‐like macrophages. The mechanism underlying this effect was then further studied. Oncogene‐driven expression of cytokines critical for the recruitment and phenotype of immune cells, particularly cells of the myeloid lineage, has been reported. Monocytes are recruited and activated mainly by tumor‐derived signals such as chemokines, cytokines, and other endogenous signals.^[^ ^39^, ^40^ ^]^ Our RNA‐seq data revealed that CCL5 is a downstream gene of ITG*β*8. CCL5 is known to be a potent chemoattractant for monocytes, T helper cells, and eosinophils.^[^ ^41^ ^]^ As reported, the expression of CCL5 in tumor cells can be upregulated by the enhancer of zeste 2 polycomb repressive complex 2 subunit (EZH2) to promote macrophage recruitment.^[^ ^42^ ^]^ CCL5 secreted by luminal B breast cancer cells binds to CCR5 to induce the polarization of macrophages toward the M2 phenotype through activation of the mitogen‐activated protein kinase kinase 7 (MEK)/STAT3 signaling pathway.^[^ ^43^ ^]^ Additionally, CCL5 can also be secreted by TAMs and activated by *β*‐chain protein/STAT3 signaling, which promotes prostate cancer cell stemness and metastasis.^[^ ^27^ ^]^ Overall, the CCL5/CCR5 axis is the primary factor involved in macrophage differentiation and tumor progression.

In our study, ITG*β*8 regulated the mRNA expression and secretion of CCL5 in LUAD cells, thereby binding to CCR5 to recruit more macrophages. Knockdown of CCL5 in A549 cells reversed the increase in the proportion of M2 macrophages induced by ITG*β*8 overexpression. Furthermore, biological function experiments revealed that CCL5 knockdown inhibited the A549‐ITG*β*8‐TCM‐mediated promotion of tumor cell proliferation, migration, and invasion. Mechanistically, we revealed that ITG*β*8 upregulates CCL5 via the PI3K/AKT/IRF9 axis. Recent studies have demonstrated that IRF9 plays an important role in the development of cancer, and in breast cancer, IRF9 overexpression confers resistance to antimicrobial agents.^[^ ^25^ ^]^ In neuroblastoma (NB), polypyrimidine tract binding protein 2 (PTBP2) induces alternative splicing of IRF9 within the exon 6‐7‐8 region, leading to tumor‐associated monocyte/macrophage chemotaxis and repolarization^[^ ^44^ ^]^ In the present study, we found that IRF9 regulates CCL5 mRNA expression and secretion and is regulated by ITG*β*8/PI3K/AKT pathway. The PI3K/AKT signaling pathway is a critical pathway that regulates tumor proliferation and metastasis.^[^ ^45^ ^]^ The activation of AKT can directly or indirectly activate pro‐EMT transcription factors, stimulate the EMT process, and induce prometastatic molecules, leading to tumor metastasis.^[^ ^46^ ^]^ Notably, the overexpression of ITG*β*8 activated the phosphorylation of key PI3K/AKT proteins in LUAD cells, and treatment with LY294002 reversed the increase in CCL5 secretion observed in ITG*β*8‐overexpressing LUAD cells. Similarly, LY294002 significantly inhibited the M2 polarization of macrophages and the progression of LUAD cells induced by ITG*β*8. In vivo, we observed the upregulation of IRF9, p‐PI3K, p‐AKT, and CD163 in subcutaneous tumor tissues from nude mice injected with ITG*β*8‐overexpressing A549 cells. In addition, ITG*β*8 increased the expression of CD163 and Arg1 in subcutaneous tumors of nude mice, while LY294002, shIRF9, and shCCL5 reversed the M2 polarization ability of macrophages induced by ITG*β*8 to a certain extent. Although the data show that the induction effect of ITG*β*8 is greater than the downstream blocking effect, this may be attributed to the intricate regulation within the TME. Further and in‐depth research to explore the mechanism by which ITG*β*8 regulates M2 polarization in macrophages is necessary. TGF‐*β* secreted by cancer cells is essential for the repression of the anti‐tumor immune response, with its activation being largely dependent on integrins. In ovarian cancer, integrin‐mediated NF‐κB and TGF‐*β*2 signaling pathways are involved in inducing cytokine/chemokine production in cancer cells, promoting the mobilization and differentiation of M2 macrophages.^[^ ^19^ ^]^ These factors may serve as potential mediators in the mechanism through which integrin regulates macrophage polarization, warranting further investigation in future research.

The interactions between tumor cells and immune infiltrating cells are usually mutual. Tumor cell products affect the M1/M2 transformation of TAMs, and TAMs, in turn, regulate the biological behavior of tumor cells by secreting various cytokines.^[^ ^47^ ^]^ Therefore, we next explored the feedback effect of M2‐like macrophages on LUAD cells. Most notably, we observed that, compared with the level in the control group, the protein level of ITG*β*8 was increased in LUAD cells treated with CM from M2‐like macrophages. Interestingly, we found that ITG*β*8 expression was upregulated by the addition of IL8 and IL10 but not by the addition of IL4. During this process, SPI1 was identified as an important mediator. As a transcriptional regulatory factor, SPI1 is involved in the progression of various cancers. In hepatocellular carcinoma (HCC), SPI1 serves as a downstream effector of PR/SET domain 1 (PRDM1) to increase programmed cell death 1 ligand 1 (PD‐L1) transcription, which contributes to cancer immune evasion.^[^ ^48^ ^]^ SPI1‐mediated MIR222 host gene (MIR222HG) transcription promotes the proneural‐to‐mesenchymal transition of glioma stem cells^[^ ^49^ ^]^ According to a previous study on GBM, SPI1 promotes tumor progression by inducing fat mass and obesity‐associated protein (FTO) downregulation to regulate pri‐miR‐10a processing in an m6A‐dependent manner.^[^ ^50^ ^]^ Furthermore, SPI1 might serve as a single biomarker for predicting the prognosis and metastasis risk in NSCLC patients.^[^ ^51^ ^]^ Therefore, we predicted the binding site of SPI1 and ITG*β*8 and validated the results via dual‐luciferase reporter assays. These results indicate that SPI1 mediates the induction of ITG*β*8 by M2‐like macrophages.

Overall, this study suggests for the first time that high expression of ITG*β*8 contributes to a positive feedback loop between tumor cells and macrophages that drives LUAD progression. Our results shed new light on the role of ITG*β*8 in the TME of LUAD and provide a mechanistic basis for macrophage polarization and macrophage‐induced progression in LUAD cells.

## 4 Experimental Section

### Bioinformatics Analysis

In this study, the TCGA (http://cancergenome.nih.gov), GEO (https://www.ncbi.nlm.nih.gov/geo/), UALCAN (http://ualcan.path.uab.edu), TISIDB (http://cis.hku.hk/TISIDB/), NCBI (https://www.ncbi.nlm.nih.gov/), hTFtarget (https://guolab.wchscu.cn/hTFtarget/#!/) and JASPAR (http://jaspar.genereg.net) datasets were explored. The scRNA‐seq (GSE189357) dataset was used to analyze the distribution of ITG*β*8 or CCR5 in the TME. The Kaplan–Meier plotter (KM plotter, http://kmplot.com) database was used to analyze clinical features. The GEPIA2 (http://gepia2.cancer‐pku.cn) database was used to analyze the correlation between the two genes.

### Cell Lines and Cell Culture

HBE, A549, H1299, H1975, and H1993 cells were maintained in RPMI‐1640 (Gibco, USA) supplemented with 1% penicillin/streptomycin (Beyotime, China) and 10% fetal bovine serum (FBS) (PAN, Germany). HEK293T cells were maintained in DMEM (Gibco, USA) supplemented with 1% penicillin/streptomycin and 10% FBS. The cells were obtained from the Heilongjiang Cancer Institute (Harbin, China). Human THP‐1 monocytic cells were purchased from Procell (Wuhan, China) and cultured with RPMI‐1640 supplemented with 1% penicillin/streptomycin, 10% FBS, and 0.05 mM *β*‐mercaptoethanol (Procell, China). All the cells were cultured at 37 °C in an atmosphere containing 5% CO_2_.

### Cell Transfection

ITG*β*8‐overexpressing and empty lentiviral vectors were constructed by Hanbio (Shanghai, China). Concentrated viruses were used to infect 5 × 10^5^ A549 cells in a complete medium containing 100 µL of 1 × HitransG P in a 6‐well plate. The infected cells were subsequently subjected to selection with 2 µg mL^−1^ puromycin (Biofroxx, Germany) for 72 h. To downregulate ITG*β*8, CCL5, and IRF9, siRNA (GenePharma, Shanghai, China) was transiently transfected into H1299 or H1993 cells. In particular, when the cells were grown to 30–50% confluence, scrambled siRNA (used as the negative control)/siRNA was added to the cell culture mixture via jetPRIME. Transfection efficiency was determined by qRT‐PCR and Western blotting. The target sequences are provided in Table S5 (Supporting Information).

### Differentiation of Macrophages

THP‐1 cells were seeded in a 6‐well plate at a density of 1 × 10^6^ cells/well and cultured with PMA (100 ng mL^−1^; MCE, Shanghai, China) at 37 °C and 5% CO_2_ for 48 h to promote their differentiation into adherent macrophages. Subsequently, the cells were treated with 30 ng mL^−1^ IL4 (MCE, Shanghai, China) for another 72 h to promote their polarization toward the M2 phenotype.

### Co‐Culture System

LUAD cell lines and THP‐1‐derived macrophages were co‐cultured via a cell culture insert (Corning, New York, USA) with a 0.4‐µm porous membrane to separate the upper and lower chambers. Transfected LUAD cells (5 × 10^5^ cells/well) were seeded in the upper chamber, transferred to a 6‐well plate that had previously been seeded with THP‐1 macrophages (1 × 10^6^ cells/well), and co‐cultured for 72 h.

### Cell‐Based Conditional Medium

For chemotaxis assay, LUAD‐CM from A549, H1299, and H1993 cell lines, which were subjected to ITG*β*8 overexpression or knockdown, and fresh medium (1:1) were placed in the lower chambers of the Transwell system. For the functional study of LUAD cells, TCM generated from the co‐culture system (upper: tumor cells with ITG*β*8 alteration; lower: THP‐1 macrophages) were added into the medium of LUAD cells.

### Cell Proliferation Assays

For the CCK‐8 assay, 5 × 10^3^ cells/well were seeded in a 96‐well plate and treated with TCM. Individually cultured tumor cells were used as controls. Ten microliters of CCK‐8 (Meilunbio, Dalian, China) were added to each well at 0, 24, 48, and 72 h. The cells were incubated for 1 h at 37 °C, and the absorbance values were measured at 450 nm. Each time point was assessed in replicates of at least three wells.

For the colony formation assay, 8 × 10^2^ cells/well were seeded in a 6‐well plate and fixed for 1 h after 14 days. Crystal violet was added for staining overnight. Each well was subsequently washed three times and the number of colonies was counted via Image‐Pro Plus 6.0 software.

### Cell Migration and Chemotaxis Assays

For the cell migration, 1.2 × 10^4^ cells were suspended in 200 µL of serum‐free medium, placed in the top inserts of a 24‐well Transwell plate (Corning, New York, USA), and then exposed to TCM in the lower chambers. The cells on the bottom surface of the membrane were fixed and stained with crystal violet after 24 h, and the cells that migrated were counted via Image‐Pro Plus 6.0 software.

For the chemotaxis assay, THP‐1 macrophages were seeded in the inserts, and LUAD‐CM was added to the lower chamber for 24 h.

### Wound Healing Assay

A monolayer of cells was scraped with 10‐µL pipette tips when the cells had reached 90% confluence in a 6‐well plate. Then, images were taken at appropriate time points (0 h/24 h). Image‐Pro Plus 6.0 software was used to assess the relative wound width.

### Matrigel Invasion Assay

The membrane for the invasion assay was covered with 40 µL of Matrigel (Corning, New York, USA) (diluted 1:8 with RPMI‐1640) in advance. The tumor cells were seeded in the upper chambers; the lower chambers were filled with 600 µL TCM. After 48 h of incubation, the cells that adhered to the lower filter surface were counted via Image‐Pro Plus 6.0 software.

### Flow Cytometry

THP‐1 macrophages were co‐cultured with transfected tumor cells for 72 h. Then, these macrophages were processed into single‐cell suspensions and incubated with APC‐conjugated anti‐human CD163 antibodies (Thermo Fisher, Shanghai, China) for 30 min at 4 °C. Flow cytometry was performed via a FACS Calibur flow cytometer (BD Biosciences). Flow cytometric analysis was performed with FlowJo software (FlowJo, Ashland, OR, USA).

### ELISA

The concentrations of human IL4, IL8, IL10, CCL5, and mouse CCL5 were determined strictly following the manufacturer's instructions. The ELISA kit used in the study was purchased from Boster (Wuhan, China). The absorbance was measured at 450 nm.

### qRT‐PCR

Total RNA was obtained using an E.Z.N.A. Total RNA Kit I (omega BIO‐TEK, Georgia, USA) and reverse‐transcribed to cDNA according to the manufacturer's instructions (Tiangen, Beijing, China). qRT‐PCR was conducted with an Applied Biosystems 7500 Real‐Time PCR System using FastStart Universal SYBR Green Master Mix (ROX) (Roche, Shanghai, China). The expression levels of all the targeted genes were normalized to those of GAPDH, and the fold changes were calculated via the 2^−ΔΔCt^ comparative method. The primers that were used for qRT‐PCR are listed in Table S6 (Supporting Information).

### Western Blotting

Total proteins were extracted from cells lysed with RIPA lysis buffer (Boster, Wuhan, China) containing protease and phosphatase inhibitors (Boster, Wuhan, China). The proteins were separated via 10% SDS‐polyacrylamide gel electrophoresis and transferred to polyvinylidene difluoride (PVDF) membranes. After blocking with 5% milk, the membranes were incubated with primary antibody overnight at 4 °C. HRP‐conjugated anti‐mouse or anti‐rabbit antibodies (1:20000, ZSGB‐Bio, Beijing, China) were used as secondary antibodies, and the antigen‐antibody reactions were visualized by ECL (HaiGene, Harbin, China). The primary antibodies that were used are listed in Table S7 (Supporting Information).

### Luciferase Reporter Assay

HEK293T cells were cultured at a density of 2 × 10^4^ cells/well in a 96‐well plate and transfected with the dual‐luciferase reporter construct pCDNA3.1+ITG*β*8 promoter‐WT+pRL‐tk and pCDNA3.1+ITG*β*8 promoter‐MUT+pRL‐tk or cotransfected with the luciferase reporter construct SPI1+ITG*β*8 promoter‐WT+pRL‐tk and SPI1+ITG*β*8 promoter‐MUT+pRL‐tk via Lipofectamine 2000 (Invitrogen, Carlsbad, CA) according to the manufacturer's instructions. Cell extracts were prepared at 48 h after transfection. The luciferase activity was measured with a Dual‐Luciferase Reporter Assay System (Promega, Madison, WI, USA).

### IHC

Paraffin‐embedded LUAD tissues from IV‐stage patients and mouse tumor specimen sections were used for IHC. The sections were separately incubated with anti‐ITG*β*8 (1:100, Absin, Wuhan, China), anti‐CD163 (1:1000, Proteintech, Wuhan, China), and anti‐IRF9 (1:200, Proteintech, Wuhan, China) antibodies. The scores were determined by combining the proportion of positively stained tumor cells and the intensity of staining. The proportion of positively stained tumor cells in a field was scored as follows: 0, no positive tumor cells; 1, <10% positive tumor cells; 2, 10–35% positive tumor cells; 3, 35–75% positive tumor cells; and 4, ≥75% positive tumor cells. The staining index (SI) for each sample was obtained by multiplying the intensity and proportion values. An SI ≥ 7 was considered a high expression, and samples with an SI < 7 were considered to have low expression.

### Animal

Four‐ to five‐week‐old female BALB/c nude mice were purchased from Liaoning Changsheng Biotechnology Co., Ltd. (Liaoning, China). All the experimental procedures were approved by the Ethics Committee of the Institutional Animal Care and Use Committees of Harbin Medical University. The animals were maintained under specific pathogen‐free conditions. To determine the impact of ITG*β*8 on tumor growth, A549 cells (5 × 10^6^ in 150 µL of PBS/Matrigel [1:1]) transduced with either the vector or ITG*β*8 were injected into the armpit region. The mice were sacrificed after 35 days, and the tumor weights and volumes were measured. The tumor volume was calculated via the formula (width2×lenth)/2 (mm^3^). The tumor tissues were subjected to Western blotting, H&E, and IHC staining. Additionally, apex cordis blood was extracted for ELISA.

To validate the polarization regulation of macrophages in vivo, a nude mice subcutaneous tumor model was established, collected the subcutaneous tumor, and isolated F4/80+ macrophages using the EasySep Mouse F4/80 Positive Selection Kit (STEMCELL Technologies, Canada) for qRT‐PCR. Two different animal models were used to investigate the effect of macrophages on ITG*β*8‐induced tumor growth in vivo. The macrophages in the mice were depleted via the injection of clodronate‐containing liposomes (FormuMax Scientific Inc., Sunnyvale, USA). Clodronate (200 µL) was injected into the mice 2 days before the injection of A549 cells. To prevent the repopulation of macrophages, the mice were repeatedly injected with 100 µL of clodronate liposomes every 5 days. For the subcutaneous xenograft tumor models, 5 × 10^6^ A549 cells stably overexpressing ITG*β*8 and control cells were sc injected into BALB/c nude mice. For the nude mouse metastasis model, A549 cells (5 × 10^5^ cells per mouse) were injected into the mice through the tail vein. After routine breeding and observation for 70 days, the mice were sacrificed. The lung tissues were harvested and fixed in paraformaldehyde for H&E staining.

### Statistical Analysis

Statistical analyses were conducted via the GraphPad Prism 8.0, SPSS 22.0, or R 4.3.1 programs. The statistical results were expressed as mean ± standard deviation of at least three independent experiments for each cellular group and each animal group. Student's t‐tests were used to determine statistically significant differences between groups. *p* < 0.05 was considered statistically significant (in all figures: *,*p* < 0.05; **,*p* < 0.01; ***,*p* < 0.001; ****,*p* < 0.0001; ns = not significant).

## Conflict of Interest

The authors declare no conflict of interest.

## Supporting information



Supporting Information

## Data Availability

The data that support the findings of this study are available in the supplementary material of this article.
