# Supplementary material for: Integrin β8 Facilitates Macrophage Infiltration and Polarization by Regulating CCL5 to Promote LUAD Progression
Source: Adv Sci (Weinh). 2024 Nov 13;12(2):2406865. doi: 10.1002/advs.202406865 (PMC11727125; doi:10.1002/advs.202406865)
Supplement: Supplementary file 1 — Supporting Information [file ADVS-12-2406865-s001.docx]

**Supporting Information**

**Integrin β8 Facilitates Macrophage Infiltration and Polarization by Regulating CCL5 to Promote LUAD Progression**

*Lei Song^#^, Xi Yu^#^, Yang Wu, Wenwen Zhang, Yu Zhang, Yanchi Shao, Zhenxin Hou, Chen Yang, Yue Gao*, Yanbin Zhao**

**Table S1.** Clinical characteristics of LUAD patients.

| Clinical characteristics | Numbers |
| --- | --- |
| Gender |  |
| Male | 75 |
| Female | 25 |
| Age (years) |  |
| ≤65 | 47 |
| >65 | 53 |
| Differentiation |  |
| Well/moderate | 65 |
| poor | 35 |
| T classification |  |
| T1-2 | 55 |
| T3-4 | 45 |
| Lymph node metastasis  classification |  |
| Negative | 34 |
| Positive | 66 |

**Table S2.** DEGs of RNA-seq.

| **Gene Symbol** | **log2 (ITGβ8 / Vector)** | **Qvalue (ITGβ8 / Vector)** |
| --- | --- | --- |
| TP53TG3F | 5.641778448 | 0.011362324 |
| SYNJ2BP-COX16 | 4.191799764 | 0.006571182 |
| ITGβ8 | 3.200867083 | 2.43E-218 |
| OAS2 | 2.186830774 | 2.05E-32 |
| IFITM1 | 2.134539216 | 5.27E-14 |
| MX1 | 2.065473658 | 7.14E-31 |
| IRF7 | 2.059062463 | 7.36E-75 |
| IFI27 | 2.054295289 | 5.78E-14 |
| IFI6 | 1.867926841 | 2.47E-98 |
| ISG15 | 1.853476895 | 4.76E-103 |
| CCL5 | 1.796237094 | 1.16E-19 |
| OASL | 1.603933816 | 2.31E-51 |
| BATF2 | 1.602449674 | 6.13E-11 |
| IRF9 | 1.518044384 | 8.86E-50 |
| HELZ2 | 1.451456788 | 4.32E-25 |
| IFIT1 | 1.413568119 | 8.81E-58 |
| REC8 | 1.371139202 | 2.36E-05 |
| USP18 | 1.196678186 | 9.19E-25 |
| IFIH1 | 1.18893387 | 4.70E-31 |
| DDX60 | 1.16588931 | 8.76E-19 |
| XAF1 | 1.15640659 | 0.029602979 |
| IFIT3 | 1.102307683 | 2.32E-31 |
| DDX58 | 1.036188649 | 4.03E-27 |
| RSAD2 | 1.033789459 | 0.027697807 |
| PARP9 | 1.028902242 | 5.09E-24 |
| OAS3 | 1.003104766 | 1.46E-26 |
| SAMD9 | 1.002093803 | 3.50E-19 |
| ST20-MTHFS | -1.728257515 | 8.16E-04 |
| LOC107987477 | -6.051420005 | 0.00167286 |
| SPDYE10P | -6.676571673 | 2.63E-04 |

**Table S3.** Correlation between ITGβ8 and TFs by GEPIA2.

| **TF** | **R** | **p value** |
| --- | --- | --- |
| ASCL1 | -0.095 | 0.036 |
| CEBPB | 0.022 | 0.63 |
| CTCF | 0.11 | 0.012 |
| E2F6 | 0.098 | 0.031 |
| ELF1 | 0.14 | 0.0024 |
| ETS1 | 0.23 | 1.80E-07 |
| FOXA2 | -0.0068 | 0.88 |
| LMNB1 | 0.18 | 4.50E-05 |
| MAFK | 0.018 | 0.69 |
| POLR2A | 0.053 | 0.25 |
| RAD21 | 0.057 | 0.21 |
| RB1 | 0.11 | 0.018 |
| RBL2 | 0.14 | 0.0021 |
| RELA | 0.27 | 1.50E-09 |
| REST | 0.27 | 1.20E-09 |
| SIN3A | 0.15 | 0.00067 |
| SMAD3 | 0.35 | 5.50E-15 |
| SP1 | 0.16 | 0.00052 |
| SPI1 | 0.27 | 3.10E-09 |
| TEAD4 | 0.044 | 0.34 |

**Table S4.** The sequence of ITGβ8 promoter-WT and ITGβ8 promoter-MUT in the dual-luciferase reporter analysis.

| **Promoter** | **Sequence** |
| --- | --- |
| ITGβ8 promoter-WT | AATTTCATTTCCTCTCATTA |
| ITGβ8 promoter-MUT | AATTTCCCCCAAGCTCATTA |

**Table S5.** Information of target sequences used in the study.

| **siRNAs** | **sense（5′-3′）** |
| --- | --- |
| siNC | 5′- UUCUCCGAACGUGUCACGUTT -3′ |
| siITGβ8#1 | 5′- CCAAGCUACUUGAGAAUAUTT -3′ |
| siITGβ8#2 | 5′- GCUGCAAACCUCAAUAAUUTT -3′ |
| siCCL5 | 5′- CUCAUUGCUACUGCCCUCUTT -3′ |
| siIRF9 | 5′- GCCAUACUCCACAGAAUCUTT -3′ |
| siSPI1 | 5′- CCCUAUGACACGGAUCUAUTT -3′ |

**Table S6.** Information of primers used in the study.

| **Oligo Name** | **Sequence（5′-3′）** |
| --- | --- |
| GAPDH qF | 5′-cacccactcctccacctttga-3′ |
| GAPDH qR | 5′-accaccctgttgctgtagcca-3′ |
| ITGβ8 qF | 5′-catatcggatggcgaaaagag-3′ |
| ITGβ8 qR | 5′-ccgtcattgggcaccactat-3′ |
| CD68 qF | 5′-aaggtccagggaagctgtga-3′ |
| CD68 qR | 5′-gaggtcctgcatgaatccaaa-3′ |
| CD86 qF | 5′-gcggcttttatcttcacctttc-3′ |
| CD86 qR | 5′-gttgcgaggccgcttct-3′ |
| iNOS qF | 5′-ggctgccaagctgaaattga-3′ |
| iNOS qR | 5′-tccttcttcgcctcgtaagg-3′ |
| CD163 qF | 5′-gtgcagaaaaccccacaaaaa-3′ |
| CD163 qR | 5′-cgaaaatggccaacagaaca-3′ |
| CD206 qF | 5′-gctactggaagacagcacattgc-3′ |
| CD206 qR | 5′-ggaatccatgctgtgtgatctg-3′ |
| Arg1 qF | 5′-cagtttggcaattggaagca-3′ |
| Arg1 qR | 5′-cacttgtggttgtcagtggagtgt-3′ |
| CCL5 qF | 5′-tgcatctgcctccccatatt-3′ |
| CCL5 qR | 5′-ggagcacttgccactggtgta-3′ |
| CCR5 qF | 5′-gccctgccaaaaaatcaatg-3′ |
| CCR5 qR | 5′-ggatgaggatgaccagcatgt-3′ |
| IRF9 qF | 5′-acgagtgcgtggagctcttc-3′ |
| IRF9 qR | 5′-ggcatatggccacacaggat-3′ |
| SPI1 qF | 5′-tgcaaaatggaagggtttcc-3′ |
| SPI1 qR | 5′-gtgcgtttggcgttggtata-3′ |
| mGAPDH qF | 5′-ctccactcacggcaaattca-3′ |
| mGAPDH qR | 5′-gcctcacccttgatgtt-3′ |
| mCD86 qF | 5′-tgggcttggcaatccttatc-3′ |
| mCD86 qR | 5′-tgtaaatgggcacggcagat-3′ |
| mCD163 qF | 5′-aggtgttatctgctccgagttca-3′ |
| mCD163 qR | 5′-ccatgtaccattgtaaaacacttcaa-3′ |
| mArg1 qF | 5′-tgtgtcatttgggtggatgct-3′ |
| mArg1 qR | 5′-acaggttgcccatgcagatt-3′ |

**Table S7.** Information of antibodies used in the study.

| **Reagent** | **Catalog and Source** | **Dilution** |
| --- | --- | --- |
| ITGβ8 | Cat#abs118487; Absin | 1:500 |
| IRF9 | Cat#14167-1-AP; Proteintech | 1:1000 |
| SPI1 | Cat#66618-2-Ig; Proteintech | 1:3000 |
| P-PI3K | Cat#T40065; Abmart | 1:2000 |
| PI3K | Cat#T40115; Abmart | 1:2000 |
| P-AKT | Cat#66444-1-Ig; Proteintech | 1:2000 |
| AKT | Cat#660203-2-Ig; Proteintech | 1:5000 |
| GAPDH | Cat#TA-08;ZSGB-BIO | 1:10000 |
| β-actin | Cat#220536-1-AP;Proteintech | 1:10000 |

**
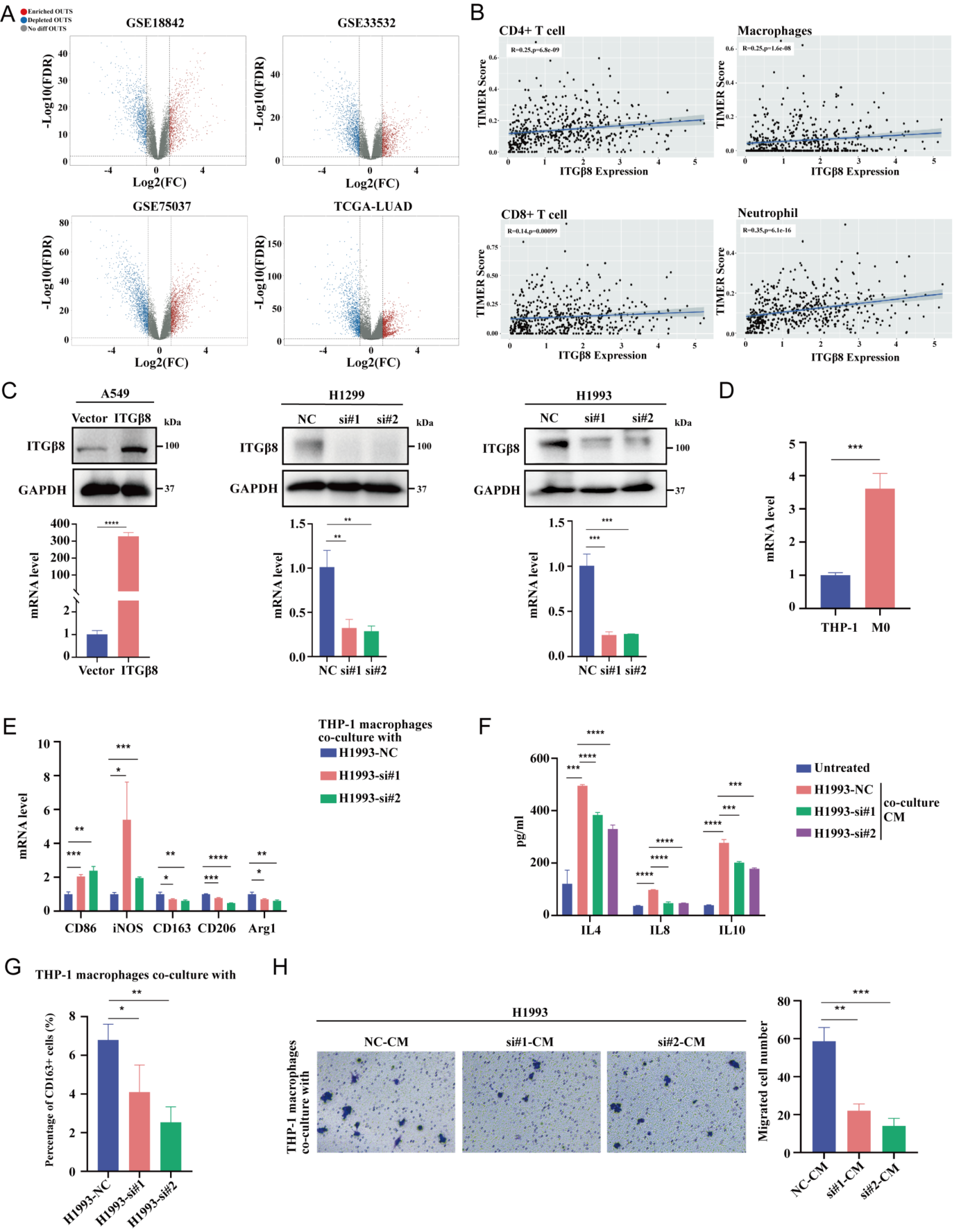
**

**Figure S1.** Knocking down ITGβ8 in H1993 cells inhibited the ability of THP-1 macrophages to polarize toward the M2 phenotype and be recruited to tumors. **A** Volcano plots of DEGs from the TCGA and GEO databases. **B** Correlation analysis of ITGβ8 and infiltrating immune cells in the TCGA-LUAD cohort via TIMER. **C** ITGβ8 protein and mRNA levels were determined in A549 cells (left panel), H1299 cells (middle panel), and H1993 cells (right panel) to confirm the effectiveness of ITGβ8 overexpression and knockdown. **D** THP-1 cells were induced to differentiate into M0 macrophages with 100 ng/ml PMA. qRT-PCR was used to quantify the expression level of CD68 in THP-1 cells. **E** qRT-PCR was used to assess the expression levels of M1-like and M2-like macrophage biomarkers in THP-1 macrophages co-cultured with H1993-siITGβ8 cells. **F** ELISA was used to measure the concentrations of IL4, IL8, and IL10 in the co-culture system. **G** Flow cytometry was used to explore the percentage of CD163+ THP-1 macrophages co-cultured with H1993-siITGβ8 cells. **H** Transwell assays were conducted to assess the effect of H1993-siITGβ8-CM on the chemotactic ability of THP-1 macrophages. (ns, not significant; * p < 0.05; ** p < 0.01; *** p < 0.001; **** p < 0.0001)

**
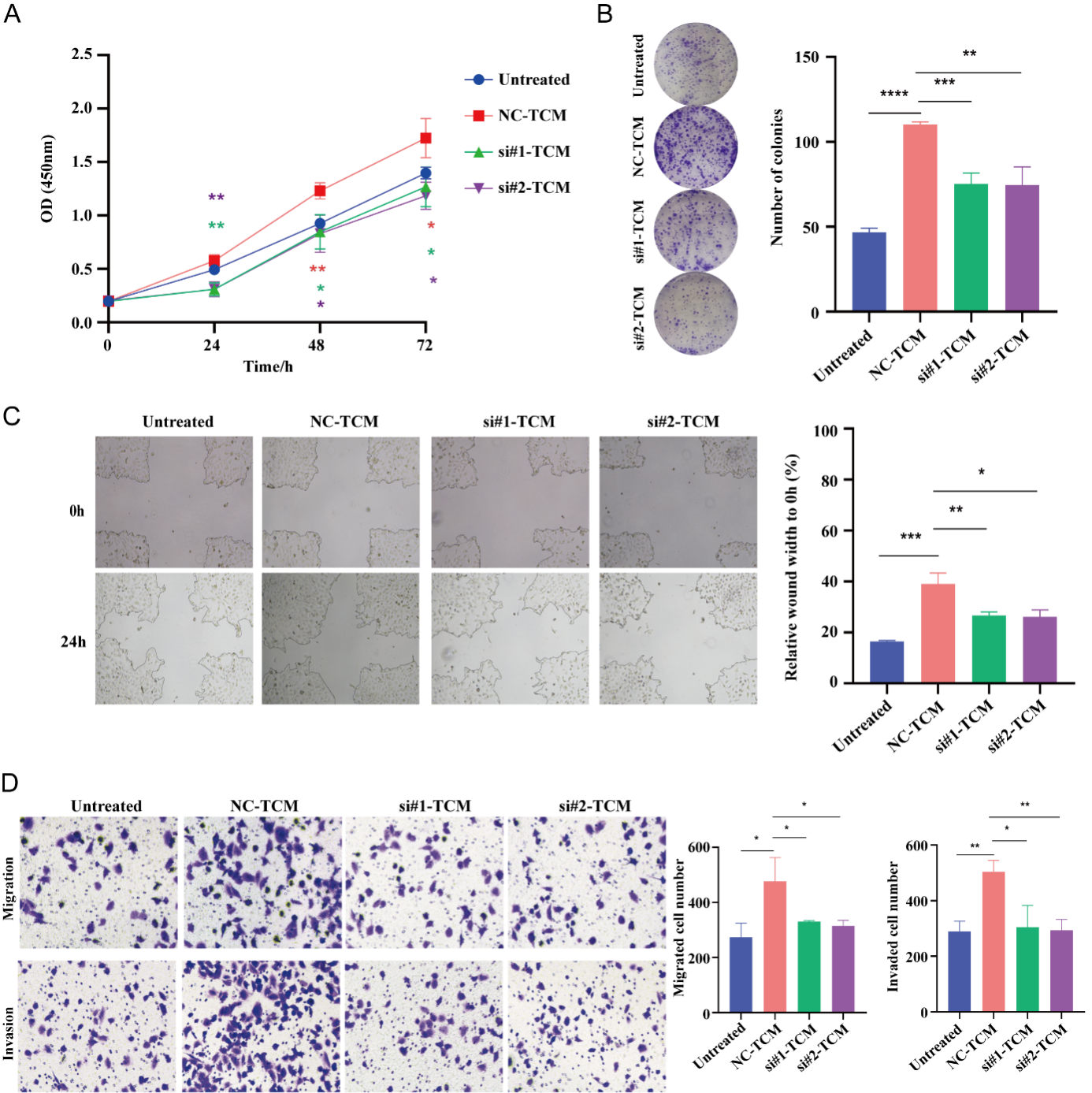
**

**Figure S2.** H1993-siITGβ8-TCM inhibited the proliferation, migration, and invasion of H1993 cells. **A, B** The effect of TCM on H1993 cell viability was analyzed via a CCK-8 assay (**A**) and colony formation assay (**B**). **C, D** The effect of TCM on the migration and invasion of H1993 cells was evaluated via wound healing assay (**C**), Transwell migration (upper panel), and invasion (lower panel) assays (**D**). (ns, not significant; * p < 0.05; ** p < 0.01; *** p < 0.001; **** p < 0.0001)


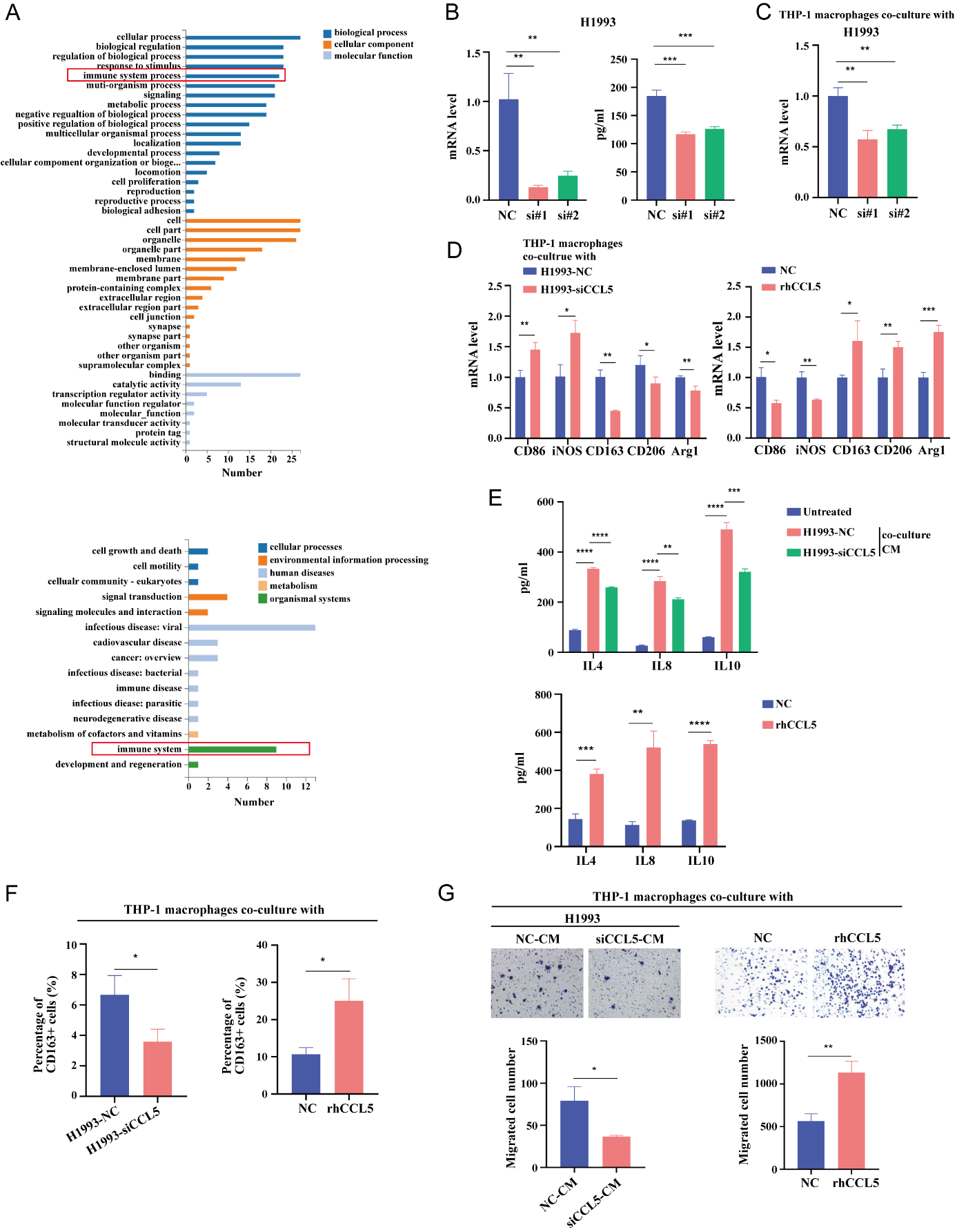


**Figure S3.** CCL5 regulates the ability of macrophages to polarize toward the M2 phenotype and promote the progression of LUAD. **A** GO (upper panel) and KEGG (lower panel) cell process functional analyses of ITGβ8 were performed via RNA-seq data. A positive relationship between ITGβ8 and immune system processes in LUAD was observed. **B** qRT-PCR and ELISA were used to quantify the regulation of CCL5 mRNA (left panel) and secretion (right panel) levels by ITGβ8. **C** Analysis of the expression of CCR5 in THP-1 macrophages via qRT-PCR. **D** M1-like and M2-like macrophage biomarker levels were measured via qRT-PCR. **E** IL4, IL8, and IL10 secretion were quantified in the co-culture system via ELISA assay. **F** Flow cytometry was used to explore the percentage of CD163+ THP-1 macrophages co-cultured with H1993 cells or rhCCL5. **G** Transwell assays were conducted to assess the effect of CM on the chemotactic ability of THP-1 macrophages. (ns, not significant; * p < 0.05; ** p < 0.01; *** p < 0.001; **** p < 0.0001)

**
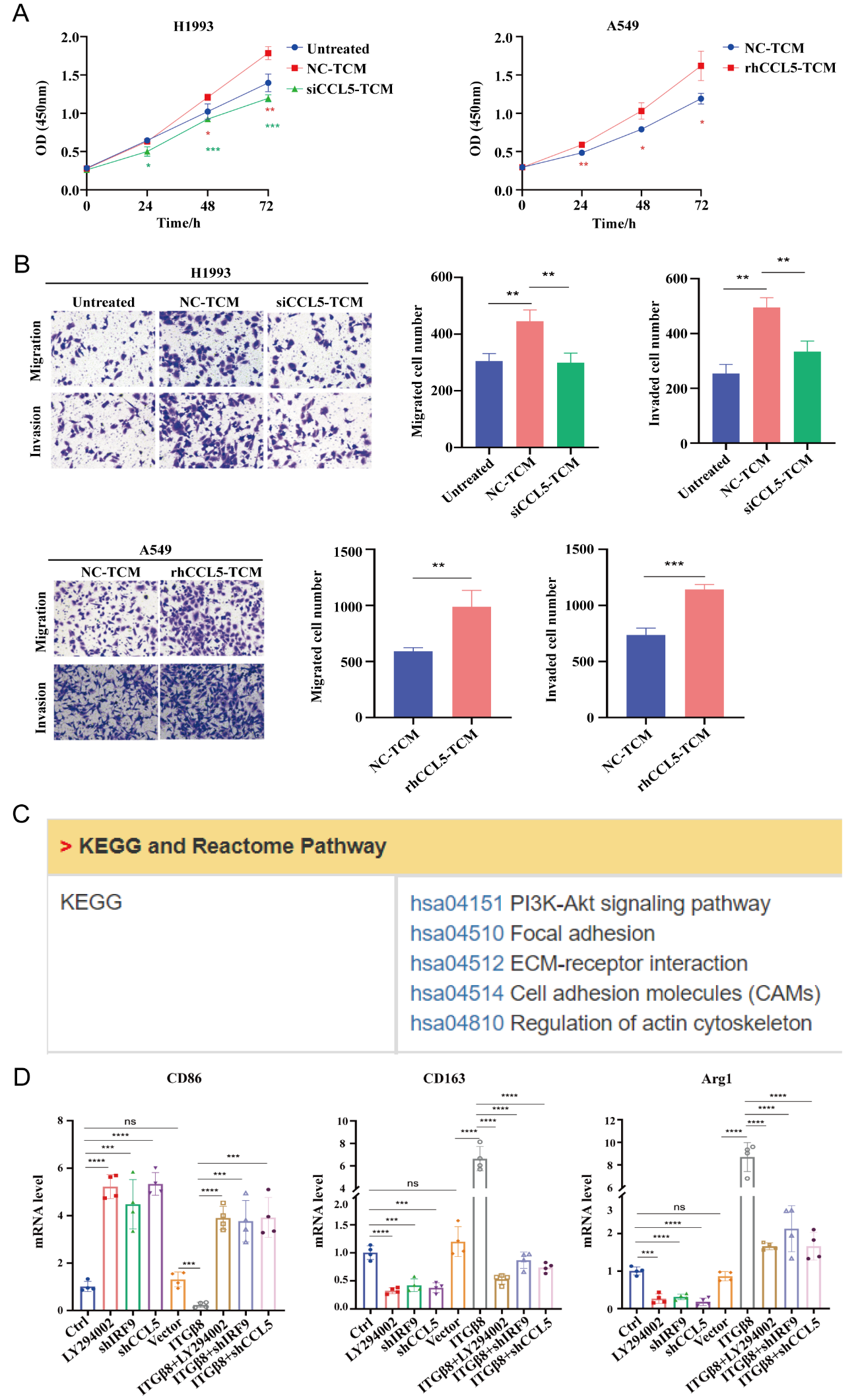
**

**Figure S4.** CCL5 regulates the proliferation, migration, and invasion of LUAD cells through macrophages. **A** The effects of TCM on the viability of H1993 and A549 cells were analyzed via CCK-8 assays. **B** The effect of TCM on the migration and invasion of LUAD cells was evaluated via Transwell migration and invasion assays in H1993 cells (upper panel) and A549 cells (lower panel). **C** Pathways related to ITGβ8 were subjected to KEGG analysis via TISIDB. **D** qRT-PCR was used to measure the expression levels of CD86, CD163, and Arg1 in TAMs isolated from the subcutaneous tumors of nude mice (n=4). (ns, not significant; * p < 0.05; ** p < 0.01; *** p < 0.001; **** p < 0.0001)
